# Understanding the Metal Distribution in Core-Shell Nanoparticles Prepared in Micellar Media

**DOI:** 10.1186/s11671-015-1048-3

**Published:** 2015-08-25

**Authors:** Concha Tojo, David Buceta, M. Arturo López-Quintela

**Affiliations:** Physical Chemistry Department, University of Vigo, E-36310 Vigo, Spain; Laboratorio de Magnetismo y Nanotecnología, Instituto de Investigaciones Tecnológicas, University of Santiago de Compostela, E-15782 Santiago de Compostela, Spain

**Keywords:** Microemulsion, Sub-nanostructure, Bimetallic nanoparticle, Simulation model, One-pot method

## Abstract

The factors that govern the reaction rate of Au/Pt bimetallic nanoparticles prepared in microemulsions by a one-pot method are examined in the light of a simulation model. Kinetic analysis proves that the intermicellar exchange has a strong effect on the reaction rates of the metal precursors. Relating to Au, reaction rate is controlled by the intermicellar exchange rate whenever concentration is high enough. With respect to Pt, the combination of a slower reduction rate and the confinement of the reactants inside micelles gives rise to an increase of local Pt salt concentration. Two main consequences must be emphasized: On one hand, Pt reduction may continue independently whether or not a new intermicellar exchange takes place. On the other hand, the accumulation of Pt reactants accelerates the reaction. As the reactant accumulation is larger when the exchange rate is faster, the resulting Pt rate increases. This results in a minor difference in the reduction rate of both metals. This difference is reflected in the metal distribution of the bimetallic nanoparticle, which shows a greater degree of mixture as the intermicellar exchange rate is faster.

## Background

A direct correlation between the surface composition of Au/Pt bimetallic nanoparticles and their catalytic activities has recently been established [1–4]. However, improving the catalytic activity in nanomaterials is a difficult task due to the complexity in controlling the surface composition. As a precise control over the nanoparticle surface arrangement is crucial to enhance catalytic activity, we have developed a simulation model to predict the metal arrangement in bimetallic nanoparticles prepared via the microemulsion route [5]. The model predictions were tested by comparison between experimental and simulation results [6]. Au/Pt nanoparticles were synthesized in a 75 % isooctane / 20 % tergitol / 5 % water microemulsion and the metal distribution was studied by HR-STEM (scanning transmission electron microscopy). Cross section with EDX analysis was performed to obtain their nanostructure. Theoretical STEM profiles were calculated from the structures predicted by simulation, using the same synthesis conditions as those of experimental studies. The successful agreement between calculated and experimental STEM profiles showed that the model is a promising tool for designing the synthesis of bimetallic nanoparticles with ad hoc controlled nanostructures.

In this paper, the prediction model is used as a tool for a better understanding of the complex mechanism governing chemical reactions in reverse micelles. As a starting point, a nanoparticle is formed from a nucleus, which can grow by building up new layers while new atoms are deposited over the previous ones. If there are two different metal atoms in the reaction medium, the metal distribution in the final particle will be defined by the sequence in the deposition of the two metals. At first, the key factor to decide this sequence is the difference in reduction potentials of both metals [5, 7–9]. That is, if ions of two different metals are very close to each other (like inside a micelle), and one metal reduces faster than the other, the nucleus and the inner layers are composed by the faster metal, and then the slower one forms the surrounding shell. In the case of both ions being reduced simultaneously, a mixture of metals is to be expected. However, core-shell geometries have been obtained from metals with quite similar reduction potentials, such as Pt/Ag [10, 11], Pd/Au [12], and Pt/Pd [13–17], so the difference in reduction potential is not the only factor.

Recently, it has been proved that another factor affecting the sequence of metals is the reactants concentration [18]. The reason is not only due to the well-known dependence of chemical kinetics on concentration but also to the compartmentalization of the reaction media. The fact that reactants are trapped inside micelles causes the accumulation of slower reduction reactants in the micelles, which favors the chemical reaction like a cage effect: if concentration of the slower reduction metal salt increases, chemical reduction will be faster, giving rise to a smaller difference between both reduction rates. As a consequence, nanoparticle structure is critically modified, because if both reductions are more simultaneous, a better mixed alloy is obtained.

In the literature, many reports have appeared describing the preparation and characterization of different bimetallic nanoparticles [19–22] and relating nanoparticle properties with microemulsion composition [23–28]. However, literature describing the metal distribution depending on microemulsion composition is rather scarce. In the research at hand, we focus on the overall rates of chemical reduction inside micelles, with the aim of improving the metal distribution in the nanostructure. The working hypothesis is that the final nanostructure is the consequence of the specific combination of three kinetic factors: the reduction rates of both metals (determined by the pair of metals), the intermicellar exchange rate (mainly determined by the surfactant), and the concentration of metal precursors inside the micelles. All of which result in a specific sequence of deposition of the metals, which in turn determines the metal arrangement in final nanoparticle. The influence of reactant concentration was previously studied [6, 18]. In this paper, we report a simulation study analyzing the influence of the intermicellar exchange rate on the synthesis of bimetallic nanostructures in microemulsions.

Because of their high catalytic activity, the nanocatalysts chosen for the study were Au/Pt [29–34]. The structure of Au/Pt nanoparticles (difference in standard reduction potentials Δε = 0.26 V), was shown to be successfully reproduced by simulation using the reduction rate ratio *v*_Au_/*v*_Pt_ ≈ 10 [6]. Because the reduction rate ratio is the simulation parameter used to characterize the nature of the metals, all results can be generalized to other bimetallic systems whose difference in standard reduction potentials is about this range.

## Methods

### Simulation Model

The kinetic course of the reaction is simulated as follows (see reference [18] for details): the microemulsion is described as a set of micelles, which move and collide with each other. Each kind of reactant (Au and Pt metal salts, and the reductor agent) is distributed through the micelles of one microemulsion. The concentration of reactants can be changed by varying the amount of reactants solved in the water phase for each microemulsion. To study the influence of concentration, we present our results using different initial averaged values of the number of reactants inside the micelles: 〈c_Au salt_〉 = 〈c_Pt salt_〉 = 〈c〉 = 4, 16 and 64 metal ions in a micelle, which correspond to 0.02, 0.08, and 0.40 M, respectively.

Brownian motion is assumed to govern the diffusion of micelles. Upon their collision, micelles can establish a water channel forming a transient dimmer, exchanging their contents (reactants, products, and/or growing particles). In each step, 10 % of micelles are randomly chosen to collide, fuse, and redisperse, allowing material exchange. One Monte Carlo step (mcs) is completed when the composition of the colliding droplets is revised after the collision, according to the criteria described below.

The intermicellar exchange protocol of free units (reactants and non-aggregated metal atoms) consists in their redistribution between two colliding droplets in accordance with the concentration-gradient principle: reactants and free metal atoms are transferred from the more occupied droplet to the less occupied one. The exchange parameter (*k*_ex_) quantifies the maximum amount of reactants (Au salt, Pt salt and/or reducing agent) and atoms (Au and/or Pt) that can be transferred during a collision. When two droplets containing different kinds of reactants collide, redistribution of the material leads to the metal salt (Au and/or Pt salts) and the reductor being located inside the same micelle. At this stage, chemical reduction can occur. The model assumes that chemical reduction of Au precursor is instantaneous (100 % of the Au salt inside the colliding micelle is reduced), and that the reduction of Pt salt is slower (only 10 % of Pt precursor is reduced). The rest of Pt salt and reducing agent remain in the micelle and will be exchanged or will react in a later collision. All atoms produced in each micelle are considered to be aggregated, forming a growing nanoparticle. As synthesis advances, more micelles simultaneously contain reactants and particles. If one colliding droplet is carrying a particle, it acts as a nucleation point, and the reaction always proceeds on it. When both colliding micelles contain particles, the reaction occurs in the micelle containing the larger particle, which has a larger surface, and so increasing the probability of it functioning as a catalyst.

The intermicellar exchange protocol of growing particles is limited by the size of the channel through which colliding droplets exchange their content; this is determined by the flexibility of the surfactant film. The flexibility parameter (*f*) specifies the size of the maximum particle for transfer between droplets. The exchange criteria for particles is also dictated by Ostwald ripening, which assumes that the largest particles grow via the condensation of material coming from the smallest particles that solubilize more readily than do the larger ones. Therefore, if both colliding droplets carry particles, the smaller one is transferred to the droplet carrying the larger ones whenever the size of the channel size is sufficiently adequate.

Despite the simplicity of the model, material intermicellar exchange protocols allow us to simulate the flexibility of the surfactant film. The following are the requirements for material intermicellar exchange to take place: the colliding micelles must remain together long enough, and the size of the channel connecting both micelles must be adequate. We can assume that the main factor determining the exchange of isolated species (reactants and free metallic atoms) is the dimer’s stability because they traverse the intermicellar channel one by one. That is, more species can be exchanged when the two micelles stay together longer (higher dimer stability), and then, channel size would not be relevant. Based on this, *k*_ex_ is related to the dimer’s stability. On the contrary, the channel size becomes a decisive factor when the transferred material is a particle made up of an aggregation of metal atoms, which must be exchanged as a whole. Consequently, this kind of material exchange will be restricted by the size of the intermicellar channel (*f*). From this picture, the flexibility of the surfactant film is included in the simulation model via the parameters *k*_ex_ (dimer stability) and the *f* (intermicellar channel size) [35]. We obtained a good agreement between the simulation and experimental results when a rigid film, such as AOT (dioctyl sodium sulfosuccinate)/*n*-heptane/water microemulsion, was related to a channel size *f* = 5, associated to *k*_ex_ = 1 free atoms exchanged during a collision [36]. Both factors must increase together, so a flexible film, such as isooctane/tergitol/water, was successful compared to simulation data using *k*_ex_ = 5, *f* = 30 [6]. In this study, we present results obtained using microemulsions with different flexibilities: rigid (*k*_ex_ = 1, *f* = 5), semiflexible (*k*_ex_ = 3, *f* = 15), flexible (*k*_ex_ = 5, *f* = 30), and very flexible film (*k*_ex_ = 15, *f* = 90).

Nanoparticle synthesis ends when the content of each micelle remains invariable. Each simulation run results in a set of micelles, where each can contain one particle, whose composition can be different. The sequence of metals of each nanoparticle is monitored as a function of time, and the results are averaged over 1000 runs. To describe the structure, the ordering of metals of each nanoparticle is stored and divided into ten concentric layers, assuming a spherical arrangement. The dispersion and averaged composition (%Au) are calculated layer by layer. The layer composition (% of each metal) is color-coded: the increase in concentration of Au goes from blue (0–10 %) to red (90–100 %). Hence, 50 % of each metal is represented by gray. As the color turns into lighter tonalities, the proportion of pure metal in the layer is higher. The histograms show the number of particles containing a given percentage of Au, monitored from the nanoparticle core (inner layer) to the shell (outer layer). In this way, the figures show how the average composition changes from the beginning of the synthesis to the end. The nanoparticle structure is also represented by concentric spheres, whose thickness is proportional to the number of layers with a given composition, keeping the same color scheme.

## Results and discussion

**Fig. 1:**
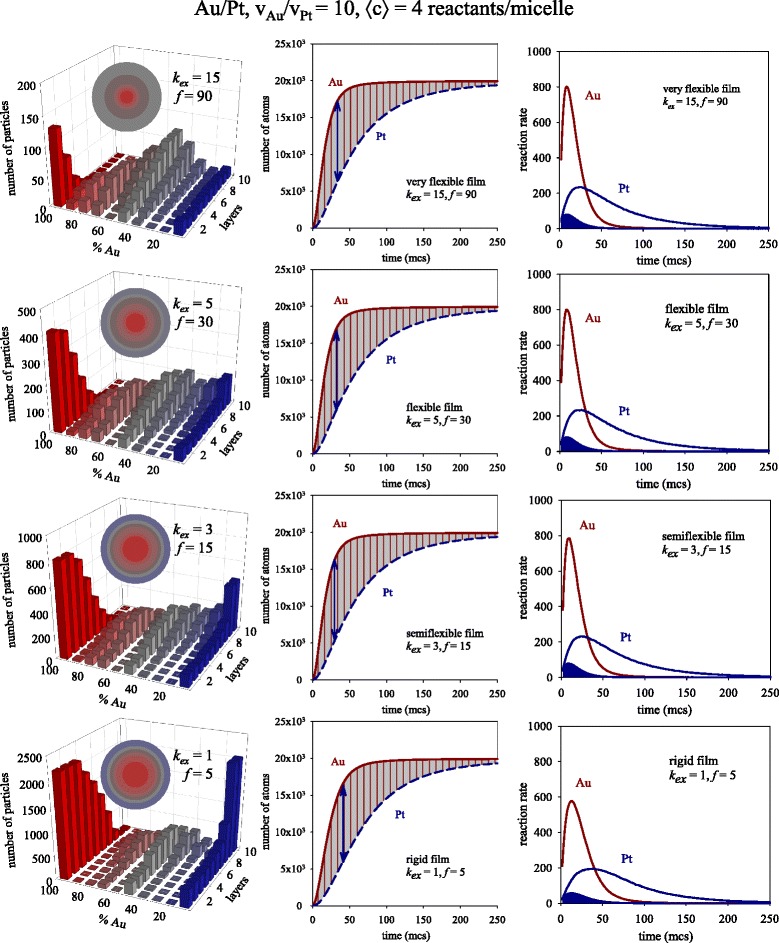
*Left column*: Predicted histograms for different values of intermicellar exchange rate keeping fixed concentration (〈c〉 = 4 metal ions in a micelle). Scheme color: *blue* (0–45 % of Au), *gray* (45–55 % of Au), *red* (55–100 % of Au). Less red means less Au (faster metal). *Colored spheres* in each histogram represent the averaged nanoparticle structure in concentric layers, keeping the same color scheme. *Center column*: Number of metal atoms obtained in micelles versus time. *Continuous* and *discontinuous lines* show the obtaining of Au and Pt, respectively. *Right column*: Reaction rate versus time. *Red* and *blue lines* show the resulting Au and Pt, respectively. *Curves* delimiting *blue areas* show the calculated Pt rate as v_Pt, calculated_ = 0.10 dn_Au_/dt

In order to illustrate the dependence of metal distribution in the final nanoparticles on intermicellar exchange rate, Fig. 1 shows the predicted nanostructures as the exchange rate parameters (*k*_ex_, *f*) decrease (see left column from top to bottom). Initial reactant concentration (〈c〉 = 4 metal ions in a micelle) and reduction rate ratio (*v*_Au_/*v*_Pt_ = 10) are kept constant. The reduction rate of Au is considered instantaneous; therefore, 100 % of pairs (Au salt and reductor) located in the same micelle give rise to Au in each Monte Carlo step (1 mcs). In the case of Pt, only 10 % of pairs (Pt salt and reductor) react. The main feature that can be drawn from this figure is the transition from an Au-core covered by alloyed layers to a core-shell structure as the intermicellar exchange rate decreases. When the exchange rate is fast (see upper figure), although the core is mainly composed by Au due to its quicker reduction rate, the high film flexibility allows for the mixture of the reactants almost from the beginning. As the intermicellar exchange decreases, the metal separation is more pronounced. On the one hand, an increasing number of particles show a pure Au core (see red bars on the left). On the other hand, the faster the intermicellar exchange, the greater the degree of alloying in the middle layers. And finally, the delay of the slower metal is enlarged by a rigid film (slower exchange rate), giving rise to higher Pt enrichment in the surrounding shell (see blue bars on the right). Previous simulation results on the influence of the film flexibility on metal segregation of bimetallic nanoparticles using different reduction rates [5] and different nucleation rates [37] lead to the same conclusion: the degree of mixture increases with the intermicellar exchange rate. It can be concluded that, even when the chemical reduction rates are different, it is possible to obtain nanoparticles with a nanoalloyed surface just by changing the microemulsion composition. This result was experimentally observed in Au-Ag [38, 39] and Au-Pt [29, 40] nanoparticles, which were prepared in alloy form if the surfactant film flexibility was high, or in a core-shell structure using a rigid film. This means that the intermicellar exchange rate, determined by the specific microemulsion, plays a key role in nanoparticle synthesis.

From a kinetic point of view, it is important to keep in mind that chemical reactivity in microemulsions is deeply affected by the fact that the reactants are trapped in micelles. Specifically, confinement of reactants inside micelles strongly affects the bimolecular electron transfer kinetics [41]. To further investigate the influence of intermicellar exchange rate on reactivity, the number of atoms of Au and Pt obtained during the synthesis are monitored as time goes on (see center column). The red and the blue lines represent the obtainment of Au and Pt, respectively. The quicker obtainment of Au as opposed to Pt is clearly observed. Likewise, both curves reach a plateau when the metal precursors have been exhausted. From the slopes of these curves, the overall reaction rate can be calculated as dn_atom_/dt, where n_atom_ is the number of atoms (Au or Pt). In this way, the reaction rate takes into account not only the chemical reduction rate (Au reduction rate is ten times faster than Pt one) but also the material intermicellar exchange rate and concentration. These calculated Au and Pt reaction rates are shown in the right column of Fig. 1 as red (Au) and blue (Pt) lines. All curves reach a maximum, from which the usual decay is obtained. The fact that the rate increases before the maximum is due to the reactants confinement inside the micelles. Only when the two reactants (the metal salt and the reducing agent) are located inside the same micelle, can chemical reduction take place. Therefore, as the reactants are redistributed between micelles, more intermicellar collisions can result in chemical reaction, giving rise to an increase of reaction rates. After the maximum, the usual decay versus time can be observed as reactants are being consumed. At first, one could expect that the reaction rate would be faster as the intermicellar exchange is faster, because a faster exchange allows a faster encounter of both reactants in the same micelle, so chemical reaction can occur. It is reflected in the slower rate reached when the film is rigid, but no meaningful difference can be observed in the other values of the intermicellar exchange rate. It is assumed to be due to the low value of concentration, because if an average number of reactants of only 4 are initially carried by each micelle, larger values of the intermicellar exchange rates (*k*_ex_ = 3, 5, 15) are irrelevant. For example, an exchange parameter *k*_ex_ = 5 implies that a maximum of 5 units of metal salt and reducing agent are allowed to be exchanged between micelles during each collision. A collision between a micelle carrying 4 reactants and an empty micelle leads to the exchange of 2 reactants from the most to the least occupied micelle (concentration-gradient criterium), so any *k*_ex_ value larger than 2 allows the exchange to take place. As a consequence, the rate profiles for *k*_ex_ = 3, 5, and 15 are similar when the averaged concentration is 〈c〉 = 4. One can assume that the overall reaction rate is limited by the concentration.

**Fig. 2:**
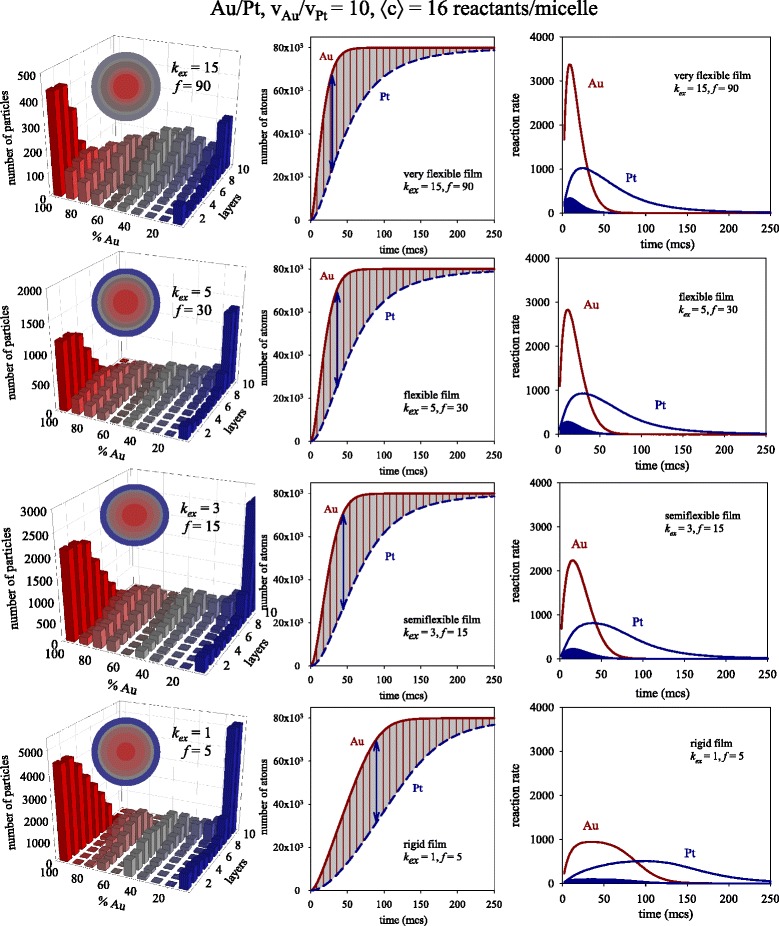
*Left column*: Predicted histograms for different values of intermicellar exchange rate keeping fixed concentration (〈c〉 = 16 metal ions in a micelle). Scheme color: *blue* (0–45 % of Au), *gray* (45–55 % of Au), *red* (55–100 % of Au). Less red means less Au (faster metal). *Colored spheres* in each histogram represent the averaged nanoparticle structure in concentric layers, keeping the same color scheme. *Center column*: Number of metal atoms obtained in micelles versus time. Continuous and discontinuous lines show the obtaining of Au and Pt, respectively. *Right column*: Reaction rate versus time. *Red* and *blue lines* show the resulting Au and Pt, respectively. Curves delimiting *blue areas* shows the calculated Pt rate as v_Pt, calculated_ = 0.10 dn_Au_/dt

**Fig. 3:**
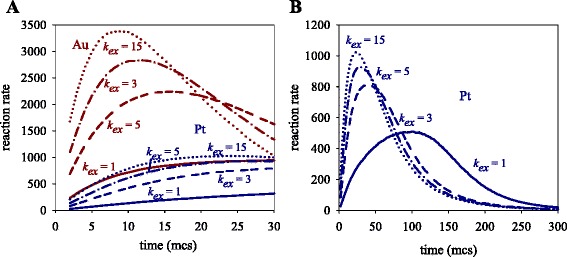
**a** Reaction rate versus time at initial stage using different values of the intermicellar exchange rate. *Red* and *blue lines* show the resulting Au and Pt, respectively. **b** Pt reaction rate versus time. Reactant concentration 〈c〉 = 16. v_Au_/v_Pt_ =10

The influence of the exchange rate on the rate profiles is expected to be higher when concentration is larger. Figure 2 shows the results obtained at larger values of initial concentration (〈c〉 = 16 metal salts). The left column shows the final nanostructures, in which the metal separation is better as the exchange rate is slower, as expected. Center column in Fig. 2 shows that, effectively, the slopes of metal obtaining curves are larger as *k*_ex_ increases, i.e., the reaction rates are significantly faster. As can be seen in the right column in Fig. 2, which shows the overall rate profiles, both Au and Pt curves are strongly dependent on the exchange rate (concentration and reduction rate ratio are kept constant). By comparing the Au and the Pt rate profiles, two points must be noted: in reference to Au, whose reduction rate is considered to be instantaneous, the fact that reactants are trapped inside micelles causes the Au reduction to be mainly controlled by the intermicellar exchange rate. To clearly demonstrate this behavior, Fig. 3 shows the reaction rates at the beginning of the synthesis for different *k*_ex_ values. Before the peak, while material is being exchanged between micelles, Au reaction rate increases faster as the intermicellar exchange is faster (see red lines in Fig. 3a), giving rise to a progressively higher maximum. Likewise, after the peak, reactants are consumed faster as *k*_ex_ is faster (note that red lines are crossed after the peak). When the exchange rate is very slow, Au reaction rate seems to reach a threshold from which it cannot increase anymore (see continuous red line). In this situation, the exchange rate is the controlling step, so Au reaction rate remains constant as long as the amount of Au salt is large enough. It is important to point out that an intermicellar control cannot be reached at a lower concentration due to Au salt being earlier exhausted (see Fig. 1).

In relation to Pt reaction rates, at first the behavior must be similar to the Au rate, because exchange restrictions are the same for both metals. However, by comparing red and blue lines in Fig. 3a, one can observe that the Au curves at different *k*_ex_ appear clearly separated, while the Pt curves are closer to each other. That is, the effect of changing *k*ex on the Pt rate seems to be much smaller than on the Au rate. This is an unexpected result, which could be explained by the effect of confinement on both metals: in the case of Au, once the intermicellar exchange allows both reactants to be carried by the same micelle, the reaction is very quick. But Pt reduction is slower, so that the reactants which failed to react the first time stay close together inside the micelle for longer. As a result, two main consequences must be noted: first, if the two reactants are carried by the same micelle, chemical reaction can take place later, regardless of whether or not a new intermicellar exchange takes place. That is, the dependence of the Pt chemical reduction on the intermicellar exchange is clearly minimized, as reflected in the approximation of Pt rate profiles (see Fig. 3b) compared to Au rate profiles.

Second, the remaining Pt reactants give rise to a local accumulation inside micelles as in a cage-like effect [18, 42, 43]. This increase in reactant concentration results in a faster Pt reaction rate. In order to understand how the kinetics of bimetallic nanoparticle formation is affected by a change in *k*_ex_, one must keep in mind that no Au salt accumulation takes place. On the contrary, the Pt rate depends on the exchange rate (like the Au rate) as well as on the accumulation of reactants, which will be higher as the exchange rate is faster (see Fig. 3b). Consequently, the difference between Au and Pt reaction rates will be expected to be smaller when the exchange is faster. By comparing the center column of Fig. 2 from the top to the bottom, it can be observed that Au and Pt curves are closer as *k*_ex_ increases (compare shaded areas). This means that, for a given value of *k*_ex_ and concentration, Pt is more accelerated by *k*_ex_ than Au.

**Fig. 4:**
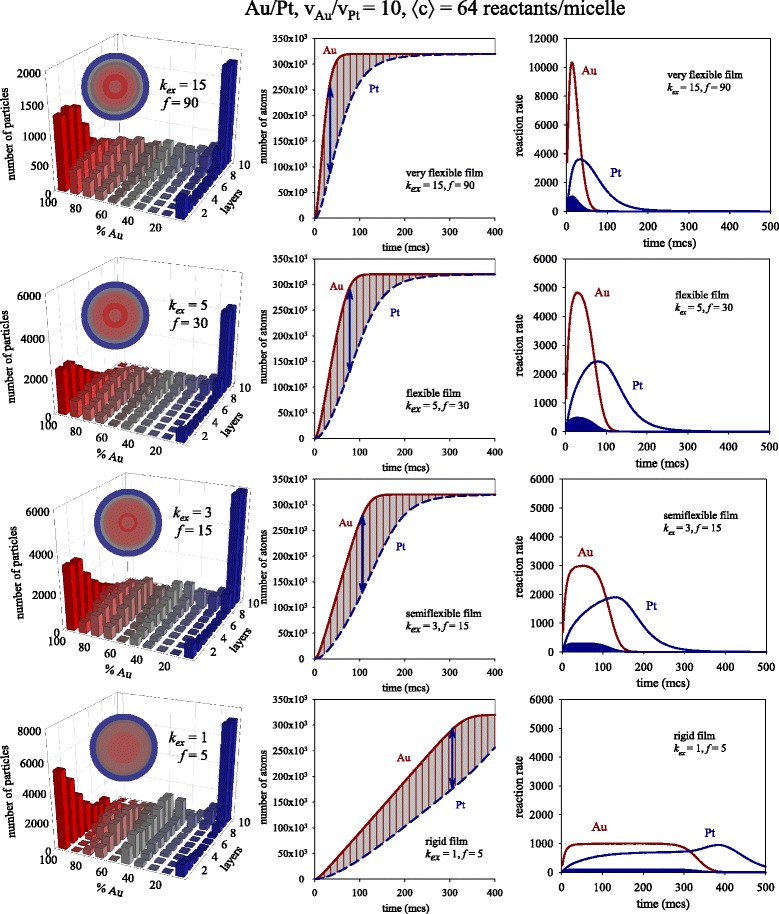
*Left column*: Predicted histograms for different values of intermicellar exchange rate keeping fixed concentration (〈c〉 = 64 metal ions in a micelle). Scheme color: *blue* (0–45 % of Au), *gray* (45–55 % of Au), red (55–100 % of Au). Less red means less Au (faster metal). *Colored spheres* in each histogram represent the averaged nanoparticle structure in concentric layers, keeping the same color scheme. *Center column*: Number of metal atoms obtained in micelles versus time. *Continuous* and *discontinuous lines* show the obtaining of Au and Pt, respectively. *Right column*: Reaction rate versus time. *Red* and *blue lines* show the resulting Au and Pt, respectively. Curves delimiting *blue areas* shows the calculated Pt rate as v_Pt, calculated_ = 0.10 dn_Au_/dt

As the cage effect has been proved to be more pronounced at higher concentrations [18], one can expect that the impact of the exchange rate on the reaction rate would also be higher. Figure 4 shows a third set of experiments obtained at larger values of initial concentration (〈c〉 = 64 metal salts). One can observe that higher slopes and clearly closer Au-Pt curves are obtained as exchange is faster. By comparing the center columns in Figs. 2 and 4, one can see that this effect is more pronounced as concentrations are high. Blue arrows in the center column of Figs. 1, 2, and 4 mark the maximum difference between the number of Au and Pt produced. The combination of steeper slopes and closer lines as *k*_ex_ increases leads to this maximum being reached sooner as the exchange is faster. The direct consequence of a more simultaneous obtainment of both metals is a greater degree of mixture in the final nanostructure, as shown in the histograms.

Also relevant in the discussion is that the rate profiles are more modified by the variation of exchange rate when concentration is higher (compare right column in Figs. 2 and 4). Larger values of rate are reached when concentration is increased, as expected. Concerning the Au rate, it is interesting to note that the sharp peak obtained at fast exchange rate becomes flat as *k*_ex_ decreases (see Fig. 4). This means that Au rate achieves the exchange rate, so it remains invariable. As can be seen in Fig. 1 (〈c〉 = 4 reactants/micelle), Au reaction rate does not reach intermicellar control at low concentration due to the Au salt finishing earlier. In the case of 〈c〉 = 16 reactants/micelle, intermicellar control is not reached, unless the exchange rate was very slow (see rigid film *k*_ex_ = 1, *f* = 5, in Fig. 2). A further increase in concentration leads to faster exchange rate controlling the kinetics (see semi flexible *k*_ex_ = 3, *f* = 15 and rigid film *k*_ex_ = 1, *f* = 5, in Fig. 4).

Finally, to further prove the acceleration of Pt reduction in micelles, the Pt rate can be theoretically calculated as follows: taking into account that Au reduction is as fast as exchange allows, the intermicellar dynamics is reflected by the Au reaction rate (dn_Au_/dt) showed in the right column of Fig. 2 (note that the values of *k*_ex_ do not depend on the kind of exchanged metal salt). Should the cage-like effect not take place, Pt salts would not be accumulated inside micelles; therefore, the Pt rate would be ten times slower than the Au reaction rate. On this basis, a theoretical Pt rate can be calculated as v_Pt_, _calculated_ = 0.10 dn_Au_/dt. In this way, the limitations due to the intermicellar exchange (included in dn_Au_/dt) would be taken into account, but without considering Pt reactants accumulation. This estimated Pt reaction rate is represented in the right column of Figs. 1, 2, and 4 as the curve delimiting the blue filled areas. The large gap between both Pt curves (calculated and obtained from simulation) can be related to the cage-like effect.

## Conclusions

The kinetics of the intermicellar exchange determines the reaction rates of both metals composing the bimetallic nanoparticle, which in turn determines the metal arrangement of bimetallic nanoparticles synthesized in microemulsions. The impact of a change in intermicellar exchange rate on the reaction rate depends on the metal: relating to Au, reaction rate is controlled by the intermicellar exchange rate whenever concentration is high enough. With respect to Pt, the combination of a slower reduction rate and the confinement of the reactants inside micelles leads to an increase of local Pt salt concentration. As a result, two consequences must be emphasized: first, contrary to Au, Pt reduction can continue independent of whether a new intermicellar exchange takes place or not; second, the accumulation of Pt reactants accelerates the reaction. Because reactant accumulation is larger as exchange rate is faster, the resulting Pt rate increases. As a nanoparticle is built up by bringing together new layers according to the order of metal reduction, the relative reaction rates of the metals determine the metal distribution in the final nanoparticle. As a result, metal segregation in a Au/Pt bimetallic nanoparticle can be manipulated just by varying the microemulsion composition. In this way, a higher degree of mixture is obtained as the intermicellar exchange rate is faster.
